# Gut microbiome biomarkers for colorectal cancer detection: a systematic review highlighting age as a key confounder

**DOI:** 10.3389/fonc.2026.1810802

**Published:** 2026-07-06

**Authors:** Pankaj Chejara, Anders Eriksson

**Affiliations:** 1 Metrosert AS, Tallinn, Estonia; 2 Centre of Genomics, Evolution and Medicine (cGEM), Institute of Genomics, University of Tartu, Tartu, Estonia

**Keywords:** colorectal cancer, diagnostic performance, faecal samples, gut biomarkers, gut microbiome, machine learning

## Abstract

The gut microbiome is a promising source of non-invasive biomarkers for colorectal cancer (CRC), yet the diagnostic performance remains heterogeneous, and the identified key markers often have inconsistency across studies, potentially influenced by host-specific factors. To ascertain the performance obtained by gut-based biomarkers in the field and investigate the impact of host characteristics on it, we conducted a systematic review and meta-analysis. After a comprehensive literature search on PubMed and Science Direct in January 2025, 27 studies were included in the review. On average, the studies reported Area Under the Receiver-Operator Curve (AUROC) of .89 and .80 for differentiating CRC and adenoma cases, respectively. We found a negative relationship between sample size and reported AUROC, though not statistically significant but still suggesting potential overfitting effect due to small dataset size, possibly inflating detection performance. Meta-regression analyses identified age difference between cases and controls as a significant moderator of diagnostic performance (*p* = 0.009), wherein cohorts with a higher age difference demonstrated a higher AUROC; in contrast, the BMI (Body Mass Index) and female ratio of study populations did not exert a significant influence. Regarding specific CRC-associated biomarkers, notable consistency was observed across studies, with an enrichment of *Fusobacterium nucleatum*, *Parvimonas micra*, and *Peptostreptococcus stomatis* and a depletion of protective butyrate producers like *Roseburia* spp. and *Faecalibacterium prausnitzii*. These findings confirm the robust aggregate potential of gut microbiome-based biomarkers for CRC diagnosis while highlighting that host age is a critical confounder that must be integrated into future predictive models to improve generalizability and clinical translation.

**Systematic review registration:**

https://www.crd.york.ac.uk/PROSPERO/, identifier CRD42024621311.

## 1 Introduction

Colorectal cancer (CRC) is the third most common cancer and the second most deadly across the globe (1). In the EU, CRC holds the second position for cancer-related mortality and ranks as the second most diagnosed cancer (2). A significant challenge with CRC is its sporadic occurrence, which accounts for 65%-70% of cases, and its non-symptomatic nature during the early stages (3). This often leads to late diagnoses, contributing to substantial economic burdens due to the high costs of treatment and patient care which could be largely reduced if CRC was detected earlier. Notably, it can take up to 10 years for a non-cancerous polyp to develop into a malignant tumour, providing a critical window for early diagnosis and intervention (3).

In response, several countries have implemented screening programs aimed at early CRC detection. The most common biomarkers used as initial screening methods are the guaiac faecal blood test (gFOBT) and the faecal immunochemical test (FIT). However, these tests offer a sensitivity in the range 50%-70% (4). To enhance early detection, recent research has explored alternative biomarkers, notably those based on gut microbiota composition (5). Emerging evidence has indicated a prominent role of gut microbiota in the development and progression of CRC (6), highlighting its potential utility as a non-invasive diagnostic tool.

Research studies have revealed the association of specific microbiota strains with CRC presence (7, 8). For example, *Enterococcus*, *Escherichia/Shigella*, *Klebsiella*, *Streptococcus*, and *Peptostreptococcus* are found to be more enriched in CRC patients than in healthy individuals (6). The identification of such microbial signatures has laid the foundation for leveraging gut microbiota in the early detection of CRC. For instance, a multi-cohort study has investigated such differences among healthy and CRC patients and achieved promising detection performance across different populations (German, French, Austrian) (9). Furthermore, a comprehensive survey of existing studies has reported overall detection performance in the range of 0.54 to 0.89 AUROC (Area Under the Receiver-Operator Curve), and sensitivity rates from 60% to 100% for detecting stage I CRC (10).

These findings collectively strengthen the claim on the potential of gut microbiota for early CRC detection. However, the variability in gut microbiota profiles due to geographic, lifestyle, and environmental factors poses challenges for translating these research findings into clinical practice. Thus, a deeper understanding of how these factors influence microbiota-based detection is crucial.

There are some existing systematic reviews (5, 9–11) that focus on identifying faecal biomarkers for CRC diagnosis. However, these reviews largely neglect the interplay between biomarkers and patient-specific factors such as demographics (e.g., age, BMI, gender). Our systematic review aims to fill this gap by examining the utility and limitations of faecal biomarkers along with patient-specific factors, providing a nuanced assessment to inform tailored screening strategies. This paper addresses the following four research questions:

- What is the performance of state-of-the art methods in distinguishing CRC (and precancerous lesions) from healthy individuals based on gut microbiome markers?
- What is the impact of host characteristics on the risk of CRC detection?
- How do sample size, age, sex, and BMI influence the detection performance of gut-microbiome-based markers for early diagnosis of CRC?
- Which biomarkers have been identified in the current state of the art and how those varies across different cohorts?

## 2 Methods

### 2.1 Search strategy

We used the widely adopted Preferred Reporting Items for Systematic Reviews and Meta-Analyses (PRISMA) methodology (12). The review protocol for this study was previously registered with PROSPERO (13). We searched for relevant research studies on PubMed and Science Direct platforms using the search query *“((gut microbiome) OR (gut microbiota) OR (gut) OR (gut bacteria) OR (gut microorganism) OR (gastrointestinal tract microorganisms) OR (GI tract bacteria)) AND ((colon cancer) OR (colorectal carcinoma) OR (colorectal cancer) OR (CRC)) AND ((early detection) OR (early diagnosis) OR (prediction) OR (diagnosis)) AND (biomarkers)”*. We included papers published in English language, and which were using original dataset, and exploring gut-microbiome biomarkers for diagnostic purposes of colorectal cancer.

The review authors (P.C. and A.E.) performed a selection of relevant studies from the pool of papers obtained after running the search query. The selection process consisted of multiple steps of filtering. The first step involved both authors independently assessing the relevance based on title, abstract, and keywords. This step resulted in assigning a code reflecting the authors’ confidence for the inclusion of the paper for the review (e.g., Include, Borderline, Exclude). All the conflicts among authors were resolved through discussion, and the final set of papers was selected for the full-text screening.

### 2.2 Eligibility criteria

We included research papers utilizing gut microbiome for its diagnostic potential for predicting CRC (or adenoma or polyps) which were published before Jan 2025 in English language. We also included the papers which did not explore predictive modelling but rather used statistical measures to identify differences between healthy and case in terms of gut microbiota composition. The review was limited to a target population of humans.

### 2.3 Exclusion criteria

We excluded studies which were not using faecal samples for the investigation of the gut microbial community. Studies with no healthy control group were also excluded. In addition, review papers, letters, and *in vitro* studies were excluded. To enable answering of the research question on assessing the impact of host characteristics on biomarkers-based diagnosis, we also excluded studies not reporting any information or providing a very limited amount of information (either 1 or 2 attributes) on host characteristics.

### 2.4 Data extraction

We extracted information on the sample, sequencing pipeline, host characteristics, and biomarkers found. For sample information, we extracted total participants, target cases (e.g., CRC, control), cases wise participants. From the sequencing pipeline, we categorized sequencing methods largely into two categories: 16S rRNA and Whole Genome Sequencing; collected target DNA region sequenced (in case of 16S rRNA). For host characteristics, we extracted age, BMI, female ratio, obese cases, and family history alongside patient’s lifestyle (e.g., smoking, alcohol consumption, exercise, diet). We extracted host characteristics for case and control separately. For biomarkers, we extracted the name of taxa reported in the paper along with an indication over enrichment (+) or depletion (-) in target case in comparison with healthy controls.

### 2.5 Outcome measures

The diagnostic performance measures such as sensitivity, specificity, AUROC score, accuracy were captured along with their confidence interval (if reported). We also recorded the machine learning algorithm and model validation approach adopted in the paper for building gut-microbiome predictors of CRC.

### 2.6 Analysis

We aggregated reported diagnostic performance metrics (e.g., AUROC) for distinguishing colorectal cancer, adenoma, or polyps from healthy controls using descriptive summaries (mean, range). Additionally, we stratified and summarized these performance measures by country to assess geographic variation.

To address the research question on how host characteristics influence CRC risk, we performed random-effects meta-analyses using the DerSimonianLaird method (14) and calculated pooled odds ratios with 95% confidence intervals for each host characteristic. For individual studies, odds ratios were computed from 2×2 contingency tables comparing exposed versus non-exposed participants among CRC/adenoma cases and controls. We derived standard errors using the delta method (15), and assigned study weights based on the inverse variance of log odds ratios. We quantified between-study heterogeneity using the I² statistic (16), with values of 25%, 50%, and 75% representing low, moderate, and high heterogeneity, respectively.

We employed weighted least-squares meta-regression to investigate the influence of sample size, age, sex, and BMI on the detection performance of gut-microbiota-based biomarkers. For sample size effects, we built a linear model regressing logit-transformed AUROC values against sample size using inverse-variance weights. Study-level AUROC variances were estimated using the Hanley and McNeil (17) approximation. For demographic covariates (age, BMI, and female ratio), we computed three variables for each: overall study mean, mean among CRC cases, and the mean difference between cases and controls. Separate weighted least-squares meta-regressions were performed for each covariate using inverse-variance weights, similar to the one used for sample size.

To synthesize biomarkers across studies reporting at different taxonomic resolutions, we harmonized reported marker names to NCBI Taxonomy identifiers and retrieved lineage/phylogenetic information for each marker using the NCBI Taxonomy database (18). This allowed direct comparison of markers reported at different ranks (e.g., genus vs. species) by linking each reported name to its full taxonomic lineage and, when appropriate, aggregating or collapsing markers to coarser ranks (e.g., genus- or phylum-level) for pooled analysis and interpretation.

## 3 Results

**Figure 1:**
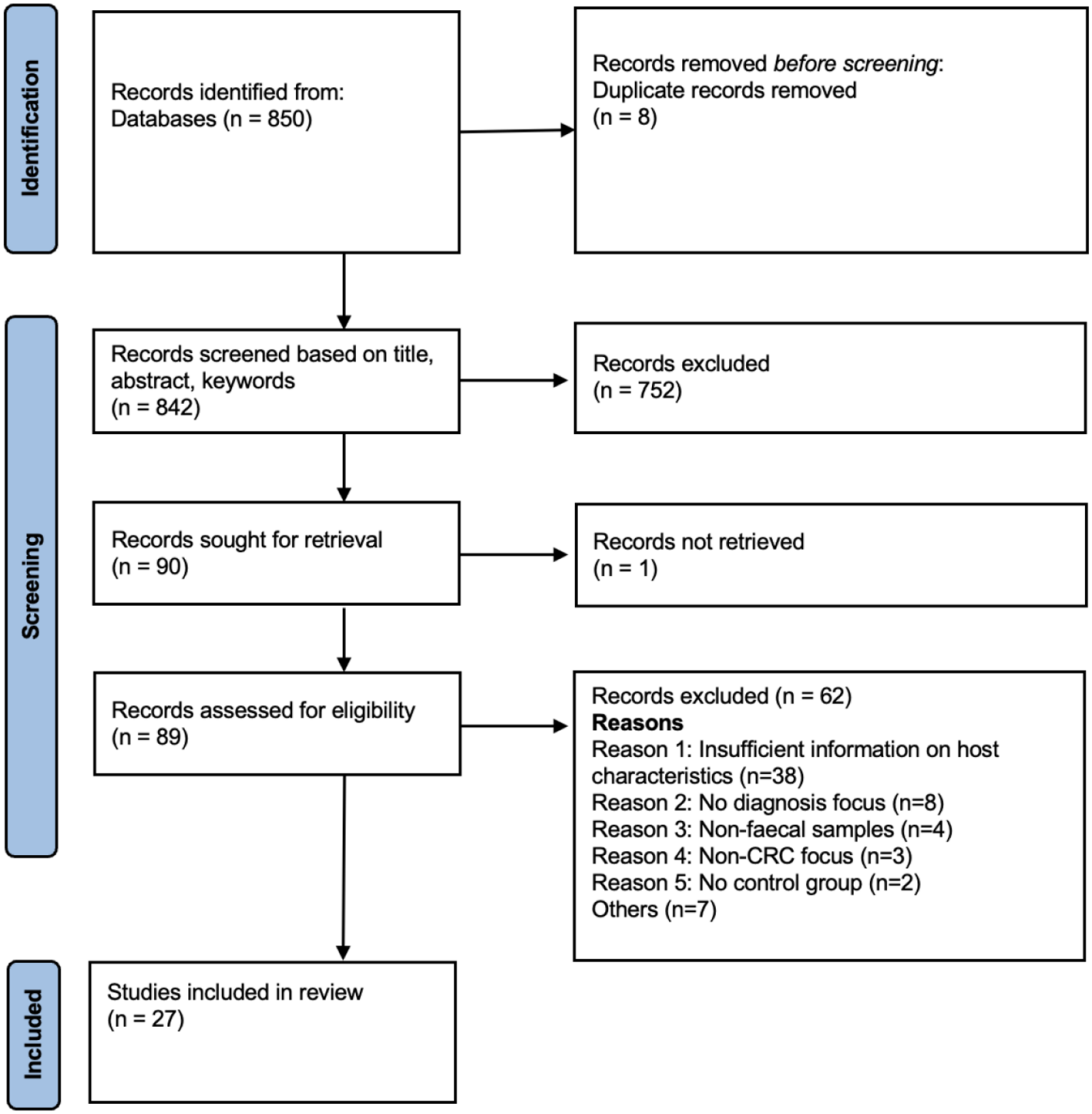
Prisma diagram.

We identified a total of 850 research papers from both platforms (**Figure 1**). We used the Rayyan platform^1^ for screening and automatically identifying duplicates (n=8). The remaining papers (n=842) were manually screened for their eligibility based on the title, abstract, and keywords, resulting in the inclusion of 90 papers for full-text screening (one article was not retrievable). The final screening step excluded 62 articles, following predefined criteria: 38 studies lacked sufficient or comparable reporting of host characteristics between groups (e.g., CRC versus controls), 8 focused on prognosis rather than case–control comparisons, 4 used non-faecal sample types, 2 did not have a control group, and the remaining 10 papers were excluded for reasons such as non-CRC focus, no original dataset, etc. After these exclusions, 27 studies met inclusion criteria and were included in the systematic review (see **Table 1** for study details).

**Table 1:** Characteristics of included studies.

Study	Country	Participants	Age (years)	Female ratio	Case definition	Sequencing	Biomarkers	ML algorithm	AUC (95% CI)
Rezasoltani et al. (19)	Iran	40 (25/15)	58/45	0.48/.33	CRC/control	16S rRNA (V3-V4)	Lachnospiraceae (+), Prevotellaceae (+), Akker mansia muciniphila (-), Coprobacillaceae (+), En terococcaceae (+), Neisseriaceae (+), Streptococ caceae (+), Bacteroides cellulosilyticus (+), Co probacillus cateniformis (+), Porphyromonas asac charolytica (+), Sphingobacterium bambusae (+), Streptococcus vestibularis(+)	SVM	0.97 (0.92-1)
Xiang et al. (20)	China	52 (26/26)	63.2/62.4	.34/.34	Advanced adenoma/control	WGS	Escherichia coli (+), Enterobacteriaceae unclassified (+), Roseburia hominis (+), Akkermansia muciniphila (+), Phocaeicola plebeius (-), Phocaeicola coprocola (-), Faecalibacterium prausnitzii (-), Roseburia inulinivorans (-), Bacteroides stercoris (-), Phocaeicola massiliensis (-), Bacteroides caccae (-), Parabacteroides merdae (-), Bacteroides eggerthii (), Dialister hominis (-)	Random forest	.79
Coker et al. (21)	China	246 (118/128)	73.2/64	.45/.52	CRC/control	WGS	Peptostreptococcus stomatis (+), Fusobacterium nucleatum (+), Parvimonas micra (+), Peptostreptococcus anaerobius (+), Bacteroides fragilis (+), Co probacter fastidosus (-), Eubacterium ventriosum (), Roseburia intestinalis (-), Roseburia inulinivorans (-), Leptotrichiabuccalis (+), Prevotella veroralis (+), Lachnospiraceae bacterium (-), Eubacterium dolichum (-)	Random forest	.9 (.87 –.93)
Coker et at. (21)	China	268 (140/128)	65.8/64	.41/.52	Adenoma/control	WGS	Leptotrichia buccalis (+), Prevotella veroralis (+), Lachnospiraceae bacterium (-), Eubacterium dolichum (-)	Random forest	.84 (.79 –.88)
Rezasoltani et al. (22)	Iran	51 (20/31)	60.8/59.8	.43/.48	CRC/control	–	Fusobacterium nucleatum (+), Enterococcus faecalis (+), Porphyromonas spp. (+), Porphyromonas gingivalis (+), Streptococcus bovis (+), Enterotoxigenic Bacteroides fragilis (+), Bacteroides fragilis (+)	Logistic Regression	.97
Gupta et al. (23)	India	60 (33/27)	59.8/41.4	.36/.63	CRC/control	WGS	Eubacterium rectale (-), Prevotella copri (-), Bifidobacterium adolescentis (-), Megasphaera elsdenii (-), Faecalibacterium prausnitzii (-), Lactobacillus ruminis (-), Akkermansia muciniphila (+), Bacteroides fragilis (+), Bacteroides clarus (+), Bac teroides eggerthii (+), Escherichia coli (+), Parvi monas micra (+), Parabacteroides distasonis (+), Flavonifractor plautii (+)	Random forest	–
Zrelli et al. (24)	Tunisia	27 (14/13)	58/59	.28/.38	CRC/control	–	Streptococcus bovis (+), Enterococcus faecalis (+), Streptococcus gallolyticus (+), Pediococcusa cidilac tici (-), Weissella confuse (-), Lactococcus garvieae (-), Enterococcus mundtii (-), Lactobacillus sakei (), Enterococcus gilvus (-), Cellulosi microbium cellulans (-), Enterococcus dispar (-), Staphylococcus equorum (-), Aerococcus viridans (-), Desemzia incerta (-), Dialister propionicifaciens (-), Weissella cibaria (-), Propionibacterium acnes (-)	–	–
Yang et al. (25)	China	107 (52/55)	53/42	.32/.52	CRC/control	WGS	Coprobacillus (+), Burkholderia (+), Porphy romonas (+), Paracoccus (+), Peptoniphilus (+), Synechococcus (+), Cyanothece (+), Roseburia inulinivorans (-), Clostridium ramosum (+), Porphyromonasgingivalis (+), F. nucleatum (+), Gemella morbillorum (+), Porphyromonas (+), Coprobacil lus (+), Burkholderia (+), Peptoniphilus (+), Rose buria inulinivorans (+), Clostridium ramosum (+), Butyrivibrio crossotus (+), Haemophilus parain fluenzae (+), Fusobacterium varium (+), Prevotella nigrescens (+), Cyanothece (+), Synechococcus (+)	Random forest	.99 (.99 - 1)
Liang et al. (26)	China	370 (170/200)	67/59	.41/.61	CRC/control	–	Fusobacterium nucleatum (+), Bacteroides clarus (), Roseburia intestinalis (-), Clostridium hathewayi (+)	Logistic regression	.88 (.85 –.91)
Fan et al. (27)	China	105 (54/51)	65/54	.37/.47	CRC/control	16S rRNA (V1-V9)	Fusobacterium (+), Bacteroides (+), Enterobacterales (+), Gammaproteobacteria (+), Proteobacte ria (+), Prevotellaceae (-), Lachnospiraceae (-), Oscillospiraceae (-), Eubacteriales (-), Clostridia (-)	Random forest	.84
Park et al. (28)	Korea	228 (70/158)	63/52.5	.35/.83	CRC/control	16S rRNA (V3-V4)	Bifidobacterium (-), Ruminococcus 1 (-), Blautia (), Eubacterium hallii (+), Odoribacter (+), Alistipes (+), Parabacteroides (+), Lachnospiraceae (-), Ruminococcaceae (+), Erysipelotrichaceae (+)	Random forest	.92
Jing et al. (29)	China	276 (92/184)	66.5/67.9	.52/.41	Advanced adenoma/control	WGS	Phocaeicola dorei (+), Prevotella sp900557255 (+), Escherichia coliD (+), Bacteroides uniformis (+), Bacteroides stercoris (+), Phocaeicola dorei (-), Escherichia coliD (-), Prevotella sp900557255 (-), CAG180 sp000432435 (-), Bacteroides uniformis (-)	CatBoost	.87
Guo et al. (30)	China	31 (11/20)	-/-	.32/.75	CRC/control	WGS	Parabacteroides johnsonii (+), Bacteroides uniformis (+), Bacteroides eggerthili (+), Lactobacillus mucosae (-), Bacteroides plebeius (-), Bacteroides coprophilus (-), Clostridium leptum (-), Coprobacter (-), Eubacterium eligens (-), Megasphaera (-)	Rambers forest	.86
Guo et al. (30)	China	34 (14/20)	-/-	.26/.75	Adenoma/control	WGS	Anaeroglobus (+), Klebsiela (+), Anaerostipes caccae (+), Lactobacillus fermentum (+), Bifidobacteriumdentium (+), Anaeroglobus geminatus (+), Bacteroides faeris (+), Bacteroides uniformis (+), Bacteroides coprocola (-), Streptococcus anginosus (-), Bacteroides massiliensis (+), Megamonas funi formis (+), Bacteroides fragilis (+), Bacteroides vulgatus (+), Coprobacter (-), Eubacterium eligens (-), Megasphaera (-)	Random forest	.66
Shi et al. (31)	China	51 (25/26)	62/60	.4/.5	CRC/control	16S rRNA (V3-V4)	Bacteroides (-), Blautia (-), Faecalibacterium (-), Corynebacterium (+), Enterococcus (+), Lactobacillus (+), Escherichia-Shigella (+), Corynebacterium (+), Enterococcus (+), Lactobacillus (+), Rom boutsia (-), Butyricicoccus (-), Parabacteroides (-), Monoglobus (-), Fusicatenibacter (-), Parasutterella (-), Roseburia (-), Faecalibacterium (-)	–	–
Gao et al. (32)	China	432 (100/332)	65.7/64.9	.39/.70	CRC/control	16S rRNA (V3-V4)	Nitrosomonas (-), Sphingomonas (-), Peptostreptococcus (+), Fusobacterium (+), Bacillus (+), Faecalibacterium (-), Ruminococcus (-), Roseburia (-), Streptococcus intermedius (+), Peptostreptococcus stomatis (+), Parvimonas micra (+), Fusobacterium nucleatum (+), Faecalibacterium prausnitzii (-), Eubacterium rectale (-), Roseburia inulinivorans (-)	Random forest	.85 (.78 -.93)
Gao et al. (32)	China	442 (110/332)	63/64.9	.37/.70	Adenoma/control	16S rRNA (V3-V4)	Bacillus cereus (+), Bacillus thuringiensis (+), Bacillus amyloliquefaciens (+), Cronobacter sakazakii (+), Nitrosomonas (-), Sphingomonas (-), Faecalibacterium (-), Ruminococcus (-), Roseburia (-), Faecalibacterium prausnitzii (-), Eubacterium rectale (-), Roseburia inulinivorans (-)	Random forest	.61 (.52 - .71)
Tesolato et al. (33)	Spain	58 (38/20)	71.2/55.8	.36/.70	CRC/control	16S rRNA (V2-V4, V6-V9)	Sutterella (-), Blautia (-), Lachnoclostridium (), Dielma (-), Olsenella (-), Compylobacter (+), Intestinimonas (+), Parvimonas (+), Turicibacter (+), Hydrogenoanaerobacterium (+), Gemella (+), Eisenbergiella (+), Peptostreptococcus (+), Ruminococcaceae (+), Lactobacillus (+), Salmonella (+), Eikenella (+), Fusobacterium (+), Escherichia-Shigella (+)	Logistic regression	.84 (.72 - .92)
Sánchez-Alcoholado et al. (34)	Spain	65 (45/20)	63.4/61.4	.5/.5	CRC/control	16S rRNA (V2-V4, V6-V9)	Prevotella (+), Clostridium (+), Desulfovibrio (+), Enterococcus (+), Bacteroides (-), Butyricimonas (), Roseburia (-), Ruminococcus (-), Alistipes (-), Victivallis (+), Enterobacter (+), Escherichia (+), Fusobacterium (+), Streptococcus (+), Blautia (-), Faecalibacterium (-), Bifidobacterium (-), Sutterella (+)	–	–
Grion et al. (35)	Spain	60 (30/30)	64/59	.3/.7	CRC/control	WGS	Ruminococcus (-), Faecalibacterium (-), Suterella (-), Eubacterium rectale (-), Prevotella (+), Segmented Filamentous Bacteria (-)	Random forest	-
Grion et al. (35)	Spain	60 (30/30)	62/59	.47/.7	Polyps/control	WGS	Bacteroides (+), Bifidobacterium (-), Dialister (-), Akkermansia (-), Parabacteroides (+), Barnesiela (+), Lachnoclostridium (+), Paraprevotella (+), Ruminiclostridium (+), Odoribacter (-)	Random forest	–
Bosch et al. (36)	The Netherlands	32 (12/20)	67/67	.50/.30	CRC/control	16S rRNA (V4)	–	Logistic regression	–
Bosch et al. (36)	The Netherlands	41 (21/20)	72/67	.15/.30	Adenoma/con- trol	16S rRNA (V4)	–	Logistic regression	–
Yu et al. (37)	China	128 (74/54)	67/62	.38/.35	CRC/control	WGS	Eubacterium ventriosum (-), Roseburia intestinalis (-), Coprococcus catus (-), Faecalibacterium prausnitzii (-), Bacteroides clarus (-), Parvimonas micra (+), Fusobacterium nucleatum (+), Solobacterium moorei (+), Peptostreptococcus stomatis (+), Clostridium symbiosum (+), Peptostreptococcus anaerobius (+)	Random forest	.96
Zeller et al. (38)	France	114 (53/61)	67/63	.45/.54	CRC/control	WGS	Fusobacterium nucleatum subsp. vincentii (+), Fusobacterium nucleatum subsp. animalis (+), Fusobacterium nucleatum subsp. nucleatum (+), Pseudoflavonifractor capillosus (+), Fusobacterium nucleatum subsp. polymorphum (+), Porphyromonas asaccharolytica (+), Eubacterium hallii (–), Eubacterium eligens (–), Prevotella nigrescens (+)	LASSO	.84
Zeller et al. (38)	France	103 (42/61)	65/63	.28/.54	Adenoma/control	WGS	Ruminococcus (-)	–	–
Zhang et al. (39)	China	55 (33/22)	59/61.4	.39/.59	Polyps/control	16S rRNA	Fusobacterium (+), Lachnoclostridium (+), Ruminococcus gnavus (+), Parabacteroides distasonis (+), Bacteroides ovatus (+), Faecalibacterium (-), Dialister (-), Faecalibacterium prausnitzii (-), Citrobacter freundii (+), Clostridium symbiosum (+), Prevotella nanceiensis (-), F. prausnitzii (-), Adlercreutzia equolifaciens (-), Ruminococcus bicirculans (-)	Random forest	.8
Hale et al. (40)	USA	780 (233/547)	66.5/66.5	.43/.43	Adenoma/control	16S rRNA	Bilophila (+), Desulfovibrio (+), Sutterella (+), Mogibacterium (+), Veillonella (-), Haemophilus (-), Firmicutes (-)	Random forest	.65
Wu et al. (41)	China	173 (121/52)	60/63	.63/.51	CRC/control	WGS	F. nucleatum (+), Fusobacterium periodonticum (+), Peptostreptococcus stomatis (+), Megasphaera elsdenii (+), Roseburia hominis (-), Anaerostipes hadrus (-), Faecalibacterium prausnitzii (-), Eisenbergiella tayiq (+), Odoribacter splanchnicus (+), Alistipes onderdonkii (+), Allisonella pneumosintes (+), CAG-83 (+), Malassezia globosa (+), Romboutsia timonensis (-), Agathobacter faecis (-), R. intestinalis (-)	LASSO	.94 (.90 -.97)
Sun et al. (42)	China	60 (30/30)	57.5/52.6	.36/.64	CRC/control	16S rRNA	Bacteroidetes (-), Erysipelotrichia (-), Flavobacteriia (-)	–	–
Sun et al. (42)	China	60 (30/30)	57.3/52.6	.43/.64	CRC metastatses/control	16S rRNA	Streptococcus (+), Butyricicoccus (+), Prevotellaceae (-), Bacteroidetes (-), Erysipelotrichia (-), Flavobacteriia (-)	–	–
Zackular et al. (43)	USA	60 (30/30)	59.4/55.3	.30/.63	CRC/control	16S rRNA (V4)	Fusobacterium (+), Porphyromonas (+), Lachnospiraceae (+), Enterobacteriaceae (+), Bacteroides (–), Lachnospiraceae (–), Clostridiales (–)	Logistic regression	.92 (.85 - .98)
Zackular et al. (43)	USA	60 (30/30)	61.3/55.3	.40/.63	Adenoma/control	16S rRNA (V4)	Ruminococcaceae (+), Clostridium (+), Pseudomonas (+), Porphyromonadaceae (+), Bacteroides (-), Lachnospiraceae (-), Clostridiales (-), Clostridium (-)	Logistic regression	.89 (.81 - .97)
Conde-Pérez et al. (44)	Spain	123 (93/30)	67.5/63.5	.38/.76	CRC/control	16S rRNA (V3-V4)	Fusobacterium (+), Parvimonas (+), Bacteroides fragilis (+), Blautia (-), Faecalibacterium sp. (-)	Logistic regression	.86
Bosch et al. (45)	The Netherlands	38 (19/19)	73/68	.10/.30	Adenoma/con- trol	16S rRNA (V4)	Butyricimonas spp. (+), Catenibacterium spp. (+), Faecalitalea spp. (+), Anaerostipes spp. (-), Bifidobacterium spp. (-), Cyanobacteria (-)	Logistic regression	.79 (.64 - .94)

^a^ Median age.

### 3.1 Performance of the state-of-the-art methods in distinguishing CRC from healthy individuals based on gut-microbiome markers

**Figure 2:**
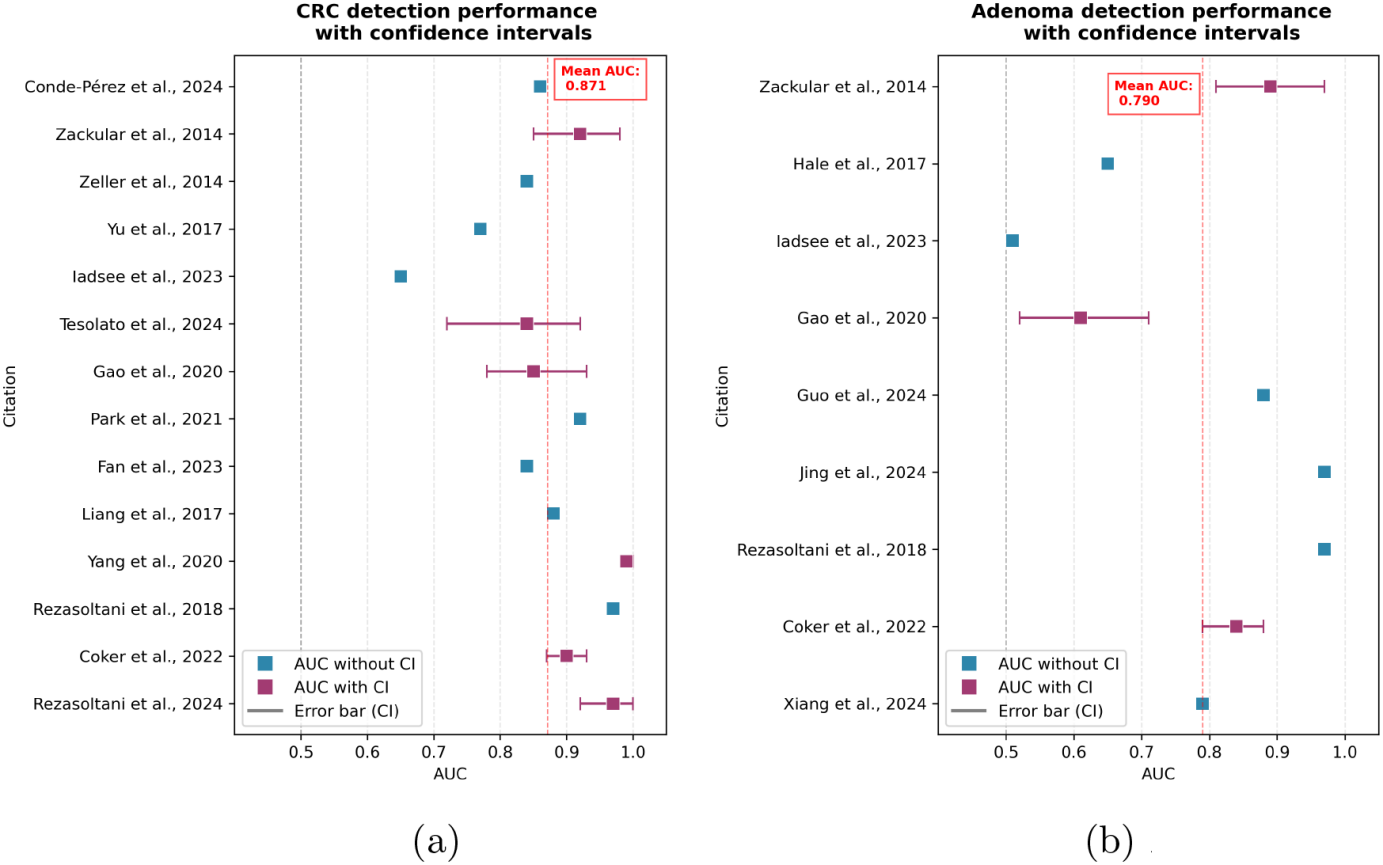
Detection performance (AUROC) across studies.

The included studies reported promising results on the potential of faecal-sample-derived gut microbiome for the detection of colorectal neoplasia. **Figure 2** shows AUROC performance metric, reported in studies for detecting CRC (**Figure 2a**) and adenoma (**Figure 2b**). Studies providing confidence interval for their performance are represented with error bars in the figures. On average, AUROC values of 0.88 and 0.79 were achieved for CRC and Adenoma, respectively. Notably, Yang et al. (25) reported achieving the highest AUROC 0.99 for differentiating CRC from healthy controls using Random Forest.

However, these performances varied widely between the studies in the range of AUROC 0.56 - 0.99. Rezasoltani et al. (19) reported achieving a score of 0.97 AUROC on a sample from Iranian population for predicting CRC. Similarly, Jing et al. (29) achieved a similar AUROC score for predicting advanced adenoma in Chinese population. While these studies demonstrated a higher prediction performance for their models, others reported a relatively lower performance measure. For example, Gao et al. (32) reported an AUROC score of 0.61 for detecting advanced adenoma in a Chinese cohort.

While most of the studies reported their detection performance using AUROC score, some studies used the accuracy metric, often alongside other metrics, for reporting their performance (n=5). For example, Jing et al. (29) reported accuracy, sensitivity, specificity, and AUROC scores for their CatBoost model to detect advanced adenoma from healthy controls. Bosch et al. (36), on other hand, reported only accuracy measures for their adenoma detection model.

**Figure 3:**
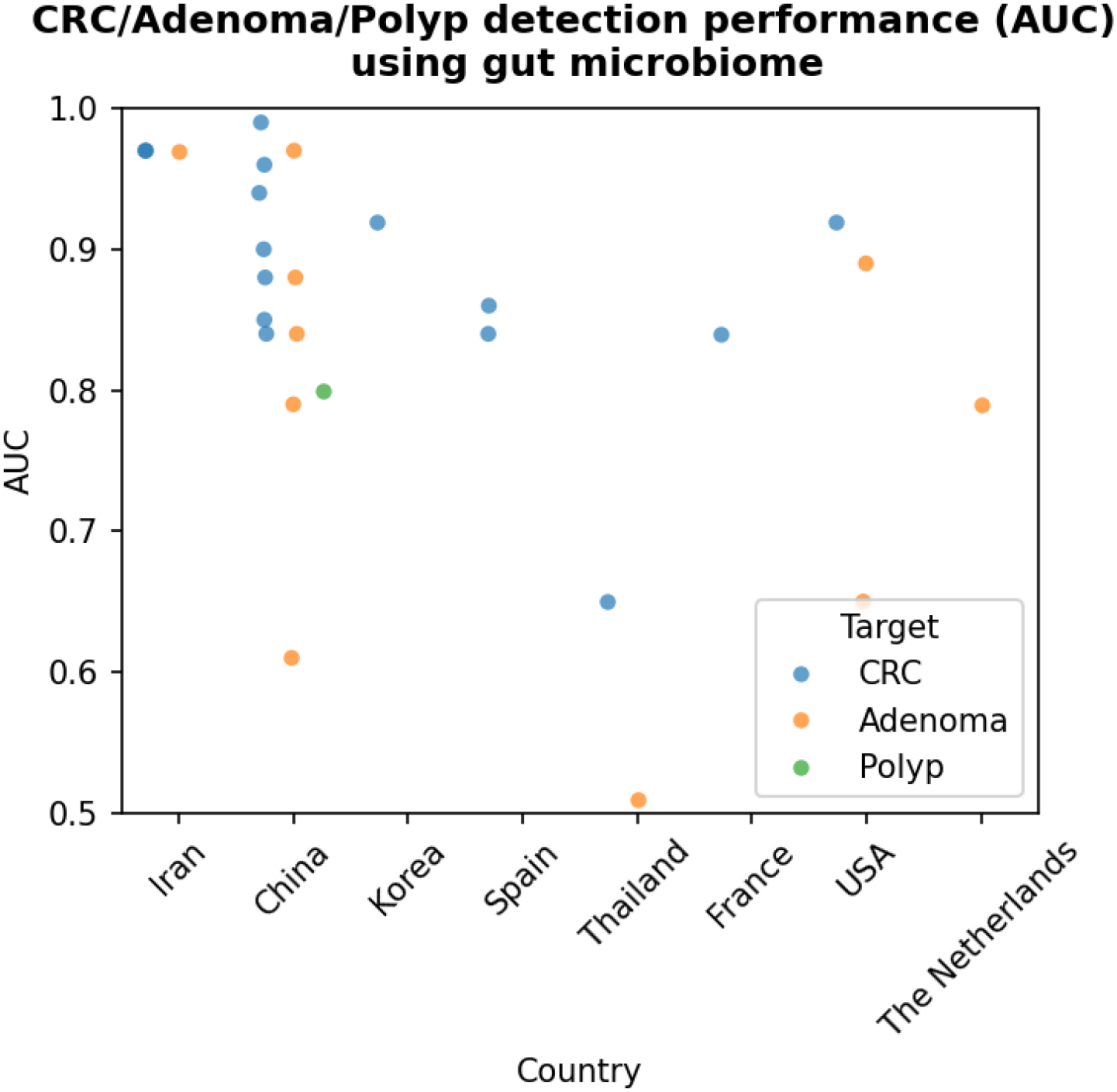
Country-wise CRC/Adenoma/Polyp detection performance across studies.

**Figure 3** displays detection performances in terms of AUROC scores reported in the reviewed studies in a country-wise manner. The plotted performance measures in the figure represent the power of differentiating target vs. control. Therefore, if a study investigated three groups (e.g., CRC, adenoma, control), then two performance measures were plotted one for each target case (e.g., CRC and adenoma) vs. control. China accounted for the largest proportion of studies, while each of the remaining countries contributed only a limited number of studies to the reviewed literature.

Researchers utilized different machine learning algorithms for building their detection model. These algorithms included Random Forest (20, 21), Logistic Regression (26, 33), LASSO (36), and Support Vector Machine (19). Most of these studies, along with others, used k-fold cross validation or train & test split methods for building and validating their models (19, 25). However, the reporting of model building approach was uncommon in the reviewed studies (i.e., 17 Studies did not report their model-building approach). Similarly, only a few studies provided information on hyperparameter tuning methods. Hyperparameters are parameters that determine a model’s structure and complexity and are not updated during training. They substantially affect model performance and therefore must be chosen carefully. Inadequate tuning methods can produce overoptimistic performance estimates: Tsamardinos et al. (46) showed that using the same cross-validation procedure for both hyperparameter selection and performance estimation inflates measured performance (a form of overfitting). Many studies’ failure to report the tuning approach prevents assessment of whether reported results are biased by such methodological shortcomings.

### 3.2 Impact of host characteristics on the risk of colorectal cancer detection

**Figure 4:**
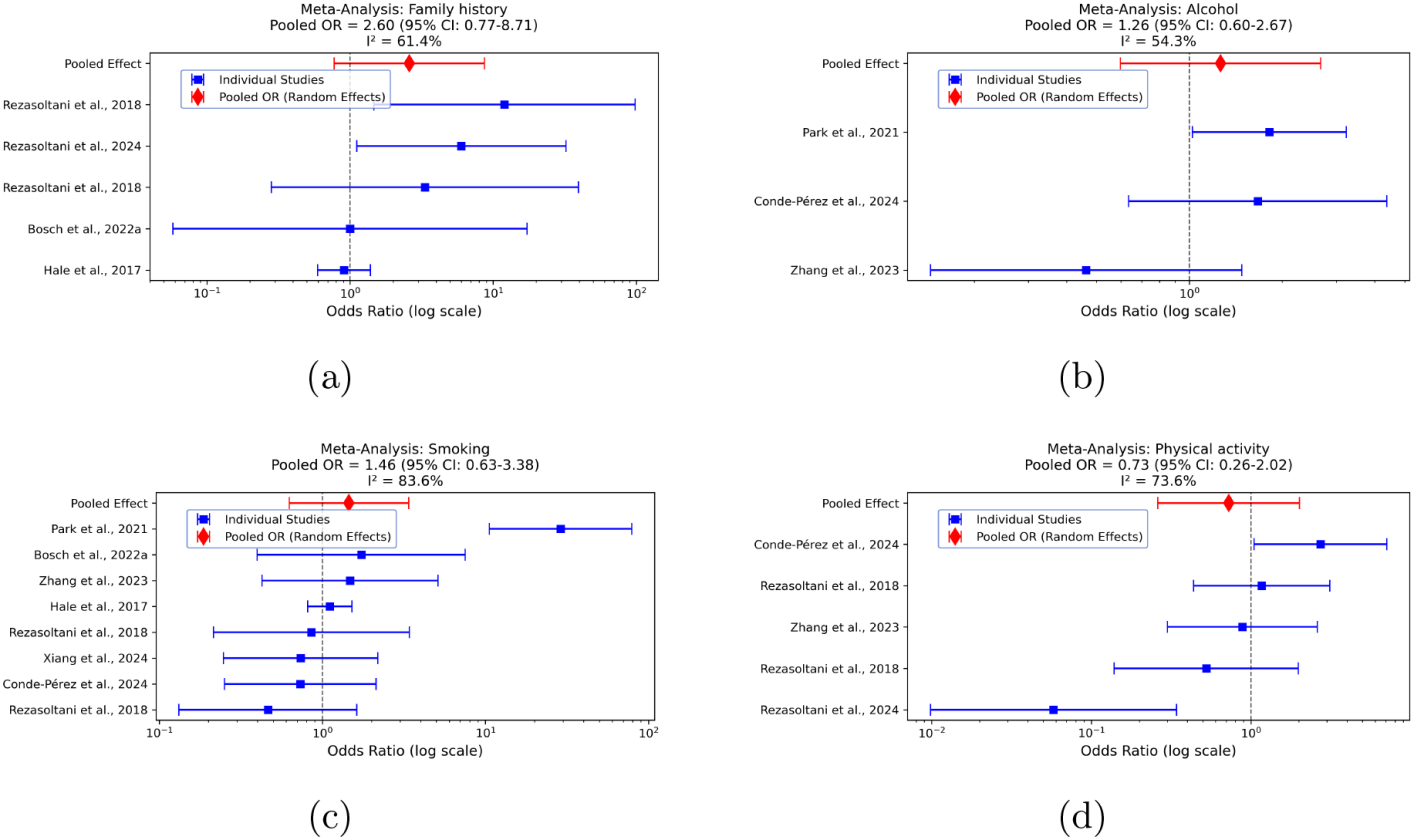
Host characteristics and risk of CRC detection.

Our meta-analyses suggested potential associations (**Figure 4**) between various host characteristics and cancer detection status, with family history showing the largest pooled effect estimate (OR = 2.60, 95% CI: 0.77–8.71). Smoking (OR = 1.46, 95% CI: 0.63–3.38) and diabetes (OR = 1.44, 95% CI: 0.72–2.89) showed elevated pooled odds ratios, while alcohol consumption demonstrated a modest positive effect estimate (OR = 1.26, 95% CI: 0.60–2.67). Physical activity showed a pooled odds ratio below unity (OR = 0.73, 95% CI: 0.26–2.02), suggesting a possible protective effect; however, none of these associations were statistically significant because all confidence intervals included 1.0.

Substantial heterogeneity was observed across studies (*I*^2^ = 54-84% for most factors) underscoring the complex interplay between host characteristics and cancer development, potentially reflecting variations in gut microbiome composition that may modify these relationships. These findings highlight the importance of considering host characteristics in conjunction with microbial biomarkers for comprehensive cancer risk assessment.

### 3.3 Impact of sample size, age, sex, BMI on gut-microbiome-based CRC diagnostic performance

**Figure 5:**
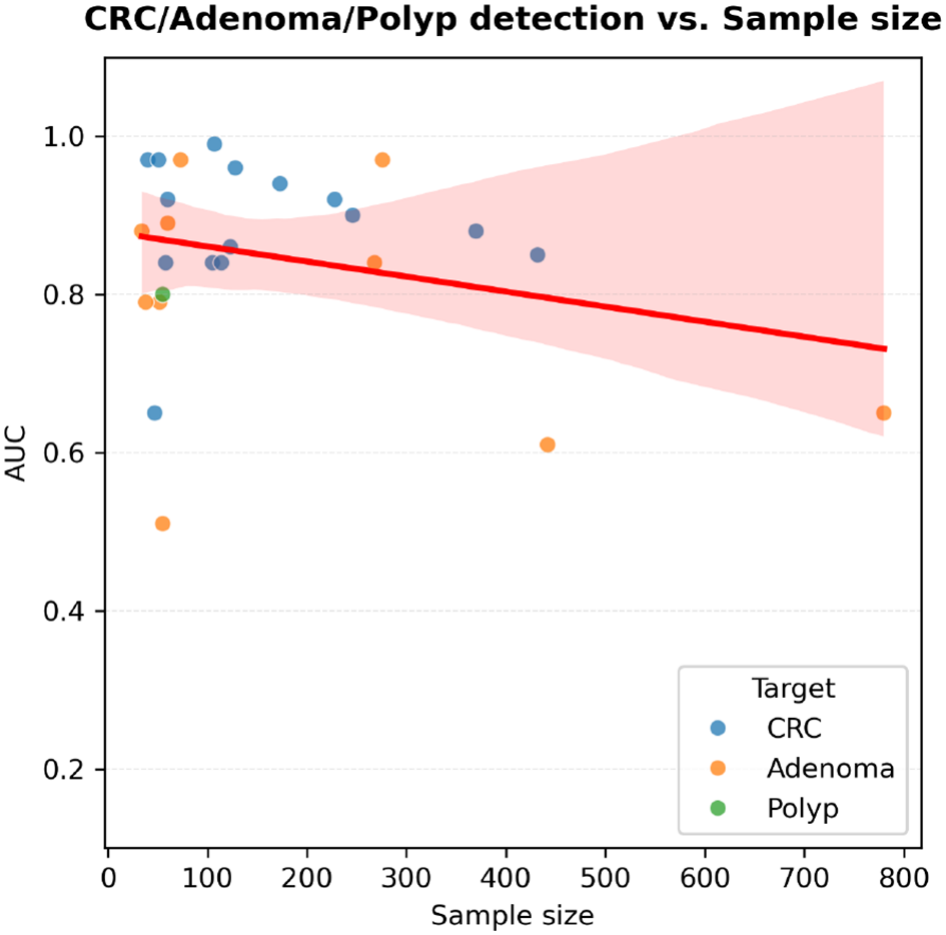
CRC/Adenoma/Polyp detection performance vs. sample size (AUC: the Receiver Operating Characteristic curve).

We noticed that a large portion of reviewed studies utilized a small sample dataset, usually less than 200 participants. **Figure 5** below shows a scatter plot between sample size and AUROC scores along with a fitted regression model. The figure indicates a potential negative relationship between sample size and AUROC scores. However, we did not find any statistically significant association between study sample size and AUROC values (*β* = -0.0002 per participant; p = 0.237). The sample size explained only 6.3% of the variance in AUROC (R² = 0.063), indicating that it is not a major determinant of reported performance.

**Figure 6:**
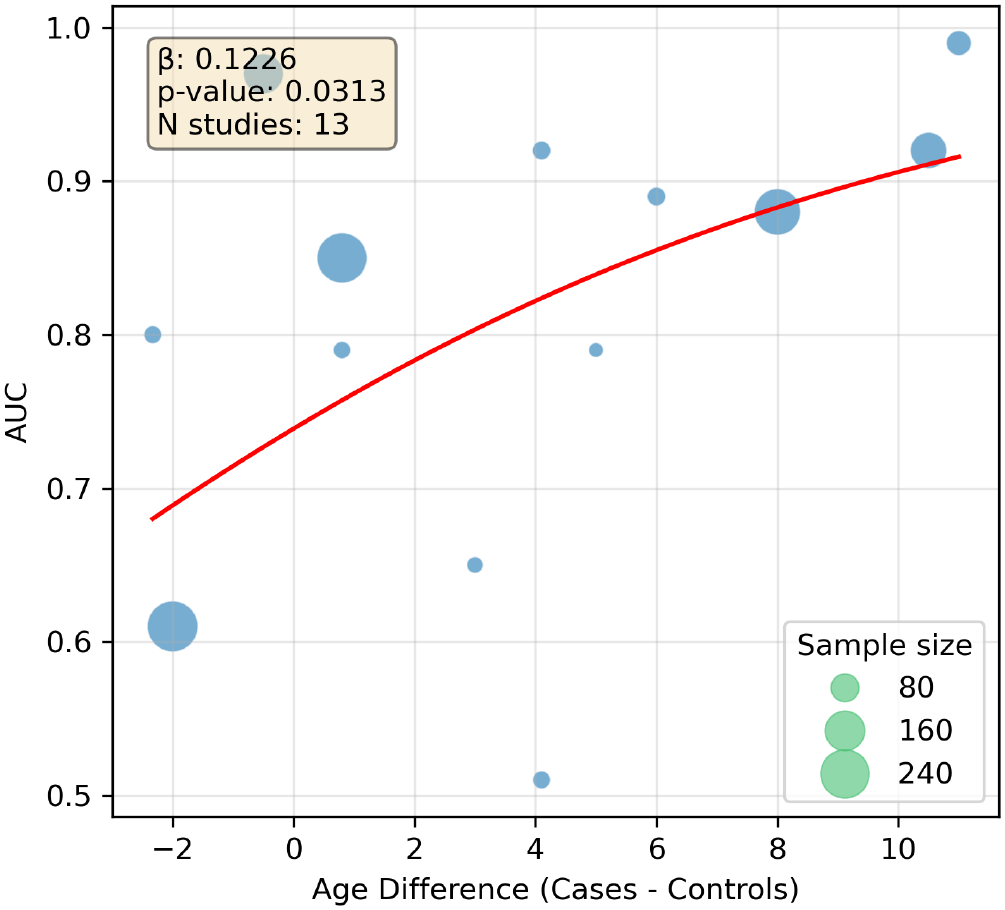
Meta regression of AUC vs. Age difference (AUC: Area Under the Receiver Operating Characteristic curve; Beta coefficient quantifies association between study-level moderator and observed effect size across included studies. In simple terms, it indicates how an unit increase in the moderator (e.g., age difference) influence the observed effect size, e.g., logit AUC).

A statistically significant association was identified between the AUROC values and the age difference between cases and controls (*β* = 0.122, p = 0.03). The meta-regression model (**Figure 6**) of age difference explained a substantial proportion of the between-study variance (R² = 0.356). The positive coefficient indicated that for every one-year increase in the mean age of cases relative to controls, the log-odds of the AUROC increased by 0.122. In more interpretable terms, studies with a larger age discrepancy between patient groups (older cases relative to controls) demonstrated significantly higher diagnostic performance. In contrast, neither the overall mean age of the study sample (*β* = -0.128, p = 0.122) nor the mean age of the CRC cases alone (*β* = 0.033, p = 0.738) were significant predictors of heterogeneity in AUROC values. The models for these covariates explained only 20.4% and 7.9% of the between-study variance, respectively, and their coefficients were not statistically significant.

No significant associations were identified between study-level BMI metrics and heterogeneity in diagnostic performance. We found that neither the overall mean BMI of the sample (*β* = 0.370, p = 0.097), the mean BMI of CRC cases alone (*β* = 0.232, p = 0.224), nor the BMI difference between cases and controls (*β* = -0.070, p = 0.770) were statistically significant predictors of the AUROC values. The overall mean BMI showed a non-significant trend and explained the most between-study variance (R² = 0.23), but ultimately, BMI-related covariates did not have a statistically significant association with diagnostic performance.

Similarly, meta-regression analysis with the female ratio of study samples revealed it as a non-significant source of heterogeneity in diagnostic performance. The overall female ratio (*β* = -4.318, p = 0.164), the ratio among CRC cases specifically (*β* = -1.346, p = 0.645), and the difference in female ratio between cases and controls (*β* = 0.593, p = 0.748) all failed to demonstrate statistically significant associations with the AUROC values. The overall ratio explained the most variance between studies (R² = 0.16), but none of the sex ratio metrics accounted for a statistically significant portion of the heterogeneity in diagnostic performance across the included studies.

### 3.4 CRC biomarkers reported in the current research and their variations across different cohorts

We identified several microbial genera which were consistently reported across studies. These were *Bacteroides* (19, 20, 29, 34), *Enterococus* (24, 34), *Faecalibacterim* (20, 23, 44), *Fusobacterium* (21, 22, 38), *Parvimonas* (23, 33), *Prevotella* (25, 35, 38), *Roseburia* (20, 22, 38), *Ruminococcus* (28, 32) and *Streptococcus* (24, 30). Certain taxa (e.g., *Fusobacterium*, *Parvimonas*) consistently showed higher abundance in colorectal neoplasia cases compared with healthy controls, whereas others (e.g., *Bacteroides*) displayed variable associations across studies.

#### 3.4.1 Country-agnostic biomarkers

Our analysis of current literature, encompassing studies from Iran, China, India, Tunisia, Korea, Spain, Thailand, The Netherlands, France, and the USA, revealed a consistent and reproducible pattern of gut microbiota dysbiosis associated with CRC and its precancerous stages. The following genera were significantly increased in CRC cohorts from at least three different countries, suggesting a strong and generalizable association with carcinogenesis.

- *Fusobacterium*: This genus was the most frequently reported oncobiome marker, identified in studies from China (21, 26, 27, 32), Iran (22), France (38), Spain (44), and the USA (43). Specific species such as *F. nucleatum* were commonly cited, underscoring its role as a key pathobiont in CRC development through mechanisms like immune modulation and pro-inflammatory signalling (47).
- *Parvimonas*: Frequently co-reported with Fusobacterium, this genus was significantly enriched in studies from China (21, 32, 37), India (23), and Spain (33, 44). Its repeated appearance highlights its potential role in the CRC-associated microbiome.
- *Peptostreptococcus*: This genus was another recurrent marker of CRC, found in studies from China (21, 32, 37, 41), India (23), and Spain (33). Species like *P. stomatis* and *P. anaerobius* were frequently reported for their association with CRC.

The depletion of certain short-chain fatty acid (SCFA)-producing genera was equally recurrent across nations, indicating a loss of protective functions in the CRC gut environment.

- *Roseburia*: This butyrate-producing genus was one of the most consistently depleted taxa across the globe. Its reduction was reported in studies from China (21, 26, 27, 32), Iran (22), India (23), and France (38). The loss of *Roseburia* spp. suggests a significant impairment of butyrate synthesis, which is crucial for maintaining colonic health and anti-inflammatory responses (48).
- *Faecalibacterium*: Another critical butyrate producer, *Faecalibacterium* (notably *F. prausnitzii*), was significantly reduced in CRC patients in studies from China (20, 27, 31, 32), India (23), Spain (34), and France (38). Its anti-inflammatory properties make its depletion a key feature of CRC dysbiosis.
- *Bifidobacterium*: This genus, known for its probiotic and immunomodulatory properties, was frequently reported to be under-represented in CRC in studies from Korea (28), Spain (34, 35), and India (23).

Our analysis also revealed several bacterial genera inconsistently associated, in terms of direction of association (enriched or depleted), with CRC and its precancerous stages across diverse geographical populations, highlighting a complex and context-dependent role of the gut microbiota in colorectal carcinogenesis.

The most prominent genera demonstrating these international, yet inconsistent associations are detailed below:

- *Akkermansia*: This genus was reported in studies from Iran (19), China (20), Spain (35), and India (23). It was significantly depleted in CRC patients in the Iranian and Spanish study but significantly enriched in both the Chinese (advanced adenoma) and Indian (CRC) cohorts. This suggests *Akkermansia*’s role may be highly dependent on sub-species, or metabolic context.
- *Bacteroides*: This group was among the most frequently reported markers, with studies from China (multiple studies), Spain, India, Iran, Korea, Thailand, and the USA. Most of the studies reported enrichment of *Bacteroides* in CRC (21, 30, 44). However, several studies from Spain (34) and the USA (43) reported a significant depletion of this genus in CRC patients compared to controls. This stark contrast underscores the profound heterogeneity within this genus, where specific species (e.g., *B. fragilis*) are strong CRC markers while others (e.g., *B. clarus*) may be protective or neutral.

## 4 Discussion

This systematic review synthesized current evidence on gut microbiome–based diagnostic performance for CRC, its associations with host characteristics (age, BMI, sex, and other demographics), and reported microbial biomarkers across geographical regions. By explicitly examining how patient-specific factors and regional variation influenced biomarker signals and diagnostic accuracy, we addressed a gap in prior reviews that primarily reported diagnostic performance without integrating host–characteristics interactions.

This review synthesized evidence from 27 studies spanning multiple countries, with reported AUROCs ranging from moderate to high, collectively supporting the diagnostic potential of stool-based microbiome biomarkers for CRC detection. Substantial methodological heterogeneity was evident across studies in sample collection and analytical procedures—sample collection setting, storage temperature and duration, DNA extraction, sequencing method and targeted region, bioinformatic pipelines, and reference databases (49). For example, Rezasoltani et al. (22) collected participant-handled samples with interim -20 ° C storage prior to transfer to -80 ° C, whereas Tesolato et al. (33) obtained hospital-collected samples stored immediately at -80 ° C.

### 4.1 CRC diagnosis using gut microbiome

The average AUROC of 0.89 indicated very high discriminative performance of gut-microbiome–based models for distinguishing CRC from controls. However, performance variability across studies, likely due to geographic and lifestyle factors and gut microbiome complexity, challenges the development of a widely applicable diagnostic model. To circumvent this limitation and improve realworld applicability, a hybrid approach integrating microbiome data with established methods appears highly promising. This is effectively illustrated by the significantly enhanced detection accuracy in studies that combine microbiome signatures with FIT (32) and with demographic factors (43).

Most studies utilized a small dataset size, usually under 200 subjects, for the development of CRC detection models. These small samples, though still valuable to gain insight into important bacterial taxa from prediction standpoint, are likely to increase the risk of inflated performance, especially when a complex model (e.g., number of model parameters is high) is used (50). This is the situation when a machine learning model fails to maintain its performance obtained with training dataset on another unseen dataset, which is termed as overfitting (51).

Many studies reported AUROCs derived from internal evaluation (same-cohort cross-validation or hold-out), rather than independent external validation; studies that did test models on external cohorts typically observed reductions in performance, underscoring limited generalizability. For example, Rezasoltani et al. (19) achieved a high score of .92 AUROC for their model on a sample size of 40. It would be a mistake to consider this score for a measure of generalizability, i.e., the model likely to achieve the same performance on the unseen dataset. Therefore, the reported performances appear promising but require thorough evaluation to move such models further from research to practice. Gupta et al. (23) developed a colorectal cancer detection model on 60 Indian samples with a test accuracy of 91%; that accuracy fell to 51.6% on a Chinese cohort and 65% on an Austrian cohort. Similarly, Yang et al. (25) reported that a model developed in Chongqing, China, dropped from an AUROC of 0.99 on the original data to 0.90 on an independent regional cohort, and further to 0.81 when applied to a Hong Kong cohort.

A current review has also highlighted concern of small dataset size in the field of oncology with the use of machine learning prediction models and recommended for larger dataset sizes (52). The observed negative trend between sample size and AUROC observed in the reviewed studies suggests that the reported high-performance measures could be due to small dataset size.

These findings imply that researchers should prioritize larger, multi-centre cohorts and rigorous external validation to obtain reliable performance estimates. Such validation approach will also enable insights into models' capabilities – in what settings the model could be used reliably and where the model will completely fail. Moreover, transparent reporting of modelling pipelines (including pre-processing, feature selection, and performance on independent test sets) will reduce bias and improve comparability.

### 4.2 Effect of host characteristics on the risk of CRC detection

Our meta-analyses involving host characteristics showed that modifiable host characteristics, particularly smoking and physical activity, may have potential associations with cancer detection status. While non-modifiable traits such as family history was found to have the strongest effect size. However, these findings should be interpreted cautiously, as they were derived from a limited number of studies for each characteristic (family history, n = 5; alcohol consumption, n = 3; smoking, n = 8; physical activity, n = 5). These findings nevertheless highlight the importance of considering host characteristics alongside microbial biomarkers in comprehensive cancer risk assessment.

Previous research studies reported significant associations of these host characteristics (53, 54). Johnson et al. (53) assessed the role of 12 CRC risk factors through meta-analysis and reported significant association of smoking, physical activity and family history. Hua et al. (54) reported these risk factors for early-onset (age *<* 50 years) CRC as well.

These findings suggest potential avenues for modulating cancer risk through lifestyle interventions, possibly mediated through gut microbiome pathways. The protective association of physical activity aligns with emerging evidence on exercise-induced microbial changes that may confer anti-carcinogenic effects (55). However, the substantial heterogeneity and wide confidence intervals observed across studies indicate that these relationships are complex and likely influenced by unaccounted confounding variables. Future research should integrate detailed microbiome profiling with host characteristic data to elucidate the mechanistic interactions underlying these associations.

### 4.3 Age as a moderator of gut biomarker-based CRC diagnosis

Our meta-regression analyses indicated that age disparities between CRC cases and controls are a substantial contributor to heterogeneity in reported diagnostic performance of microbiome-based classifiers. A greater mean age of cases relative to controls was significantly associated with higher AUROC. This pattern suggests that the discriminatory signal captured by many microbiome classifiers is, at least in part, contingent on age-related differences between groups rather than on disease status alone (56).

Previous research investigating the difference in microbial composition between early (*<* 50 years) and late (*>* 65 years) has found that microbial species varied by age between healthy and CRC samples, as well as across disease states (57). The study reported that early-onset CRCs and late-onset CRCs both had enrichment of CRC-promoting bacterial species (e.g., Bacteroides fragilis), but only late-onset CRCs were frequently found with depletion of CRC protective species (e.g., Roseburia intestinalis).

This finding has several implications. First, it emphasizes the necessity of careful design and reporting in microbiome diagnostic studies: age matching or adjustment should be routine, and performance metrics should be reported stratified by age groups to evaluate generalizability. Second, future biomarker development would benefit from mechanistic studies that disentangle age-related microbial shifts from tumour-specific signals (for example, by comparing early-stage CRC to age-matched controls, longitudinal sampling, or integrating host factors such as lifestyle), since interventions that target age-associated microbiome changes could confound diagnostic utility.

### 4.4 Gut biomarkers for CRC diagnosis

Our systematic review reveals a reproducible gut microbiome signature associated with CRC across diverse populations, characterized by expansion of pro-oncogenic/pathobiont genera and loss of protective, SCFA-producing taxa. *Fusobacterium*, *Parvimonas*, and *Peptostreptococcus* are repeatedly enriched across multiple countries, whereas *Roseburia*, *Faecalibacterium*, and *Bifidobacterium* are consistently depleted. These opposing patterns support a model in which CRC-associated dysbiosis (7) involves both gain of inflammatory, tissue-invasive bacteria and loss of butyrate-producing, homeostasis-promoting microbes.

These biomarkers are frequently reported as potential indicators for CRC. Zhou et al. (8) emphasized the roles of *Fusobacterium nucleatum* and *Bacteroides fragilis* in CRC progression, noting that *F. nucleatum* is enriched in both healthy individuals and CRC patients but increases further in fecal samples as disease advances to stage III–IV; parallel trends were observed in blood and mucosal specimens. Wu et al. (58), using multi-cohort datasets from the USA, Canada, and France, reported enrichment of Parvimonas micra, F. nucleatum, and Peptostreptococcus stomatis in CRC/adenoma groups versus healthy controls.

Genera such as *Akkermansia* and *Bacteroides* emerged as the most inconsistent with the direction of association with CRC across studies. Several reasons can be attributed to this inconsistency. First, many studies report only genus level associations, masking intra-genus heterogeneity, which is essential for distinguishing pathogenic from benign strains. Second, cross-study heterogeneity in sampling, laboratory methods (DNA extraction, 16S vs shotgun sequencing), bioinformatics pipelines, and covariate adjustment (diet, medications, BMI, age, tumour stage) introduces variability and can produce inconsistent directionality.

To address these gaps, future research should prioritize shotgun metagenomics to identify biomarkers that improve biological specificity by capturing signals across kingdoms such as Archaea, Fungi, and Bacteria. Beyond providing finer taxonomic resolution (i.e., species-level), metagenomic data enriches analyses with functional information –functional genes, metabolic pathways, and enzymatic reactions– which collectively offer an improvement over taxonomyonly-driven approaches (41).

Future research should also adopt standardised analytical pipelines to improve reproducibility and transparency and accelerate translation of research into practice. Established tools and frameworks – e.g., 16S rRNA profiling pipeline (59), MeTAline, MetaBakery for reproducible and scalable metagenomic analyses (60, 61), MAGO for the production of high-quality metagenomic-assembled genome and analysis (62)– can support this shift. Furthermore, creating common benchmarks (e.g., curatedMetagenomicData (63)) for the development, validation and comparison of biomarkers-based solutions will provide a shared baseline and facilitate a rigorous evaluation.

Standardized protocols for sampling, laboratory processing, sequencing, and analytics—combined with comprehensive covariate collection and raw-data sharing—are needed to reduce technical heterogeneity and enable pooled metaanalyses. Furthemore, future studies should also explore stratified analysis to assess independence of gut-microbiome biomarkers from host characteristics, including age and physical activity, to identify robust CRC diagnostic markers.

### 4.5 Limitations

This review has several limitations. First, the CRC detection results based on gut microbiome data should be interpreted cautiously because a number of studies were excluded for failing to report essential host characteristics or providing insufficient participant information; as a result, our synthesis is restricted to a subset of studies that evaluated machine-learning approaches for CRC detection using faecal samples. Second, there were too few studies reporting comparable host-level data to permit robust, generalizable conclusions about how host characteristics influence CRC risk. Third, our meta-regression was observational and therefore subject to confounding and bias; unmeasured factors (for example, tumor stage) may have influenced the associations we observed. Fourth, there was substantial between-study heterogeneity (*I*^2^ = 54-84%), which indicates important variation in effect estimates across studies and reduces confidence in pooled estimates; this heterogeneity, together with wide confidence intervals, reflects estimation uncertainty.

## 5 Conclusion

In this review, we showed that gut-microbiome–based biomarkers demonstrate strong aggregate discriminative performance for colorectal cancer (average AUROC 0.89), and consistently implicate pathobionts such as *Fusobacterium nucleatum*, *Parvimonas micra*, and *Peptostreptococcus stomatis*, alongside depletion of butyrate-producers (e.g., *Roseburia* spp., *Faecalibacterium prausnitzii*). Crucially, meta-regression identified age difference between cases and controls as a significant moderator, indicating that age imbalance can inflate reported diagnostic performance and may contribute to heterogeneity in estimated effects of host factors. Nevertheless, several important limitations constrain immediate clinical translation: methodological heterogeneity (sampling, storage, sequencing, bioinformatics), predominance of genus-level reporting, small and often non-representative cohorts, and reliance on internal validation. To advance clinical utility, future work should move towards implementing standardized end-to-end protocols with comprehensive covariate collection, and prioritize large, multicentre prospective cohorts with pre-specified analysis plans and rigorous external validation. Studies must also explicitly integrate and adjust for age—by including age as a covariate, stratifying analyses, or ensuring age-balanced sampling—to improve generalizability.

## Data Availability

The raw data supporting the conclusions of this article will be made available by the authors upon request, without undue reservation.

## Footnotes

1 https://www.rayyan.ai/.
